# Effects of hydrogen permeation on the mechanical characteristics of electroless nickel-plated free-cutting steel for application to the hydrogen valves of hydrogen fuel cell electric vehicles

**DOI:** 10.1371/journal.pone.0302972

**Published:** 2024-05-09

**Authors:** Dong-Ho Shin, Seong-Jong Kim

**Affiliations:** 1 Department of Marine Engineering, Graduate School, Mokpo National Maritime University, Mokpo-si, Jeollanam-do, Republic of Korea; 2 Division of Marine System Engineering, Mokpo National Maritime University, Mokpo-si, Jeollanam-do, Republic of Korea; Pakistan Institute of Engineering and Applied Sciences, PAKISTAN

## Abstract

Electroless nickel plating is a suitable technology for the hydrogen industry because electroless nickel can be mass-produced at a low cost. Investigating in a complex environment where hydrogen permeation and friction/wear work simultaneously is necessary to apply it to hydrogen valves for hydrogen fuel cell vehicles. In this research, the effects of hydrogen permeation on the mechanical characteristics of electroless nickel-plated free-cutting steel (SUM 24L) were investigated. Due to the inherent characteristics of electroless nickel plating, the damage (cracks and delamination of grain) and micro-particles by hydrogen permeation were clearly observed at the grain boundaries and triple junctions. In particular, the cracks grew from grain boundary toward the intergranualr. This is because the grain boundaries and triple junctions are hydrogen permeation pathways and increasing area of the hydrogen partial pressure. As a result, its surface roughness increased by a maximum of two times, and its hardness and adhesion strength decreased by hydrogen permeation. In particular, hydrogen permeation increased the friction coefficient of the electroless nickel-plated layer, and the damage caused by adhesive wear was significantly greater, increasing the wear depth by up to 5.7 times. This is believed to be due to the decreasing in wear resistance of the electroless nickel plating layer damaged by hydrogen permeation. Nevertheless, the Vickers hardness and the friction coefficient of the electroless nickel plating layer were improved by about 3 and 5.6 times, respectively, compared with those of the free-cutting steel. In particular, the electroless nickel-plated specimens with hydrogen embrittlement exhibited significantly better mechanical characteristics and wear resistance than the free-cutting steel.

## 1. Introduction

Hydrogen fuel cell electric vehicles (FCEVs) have various advantages over conventional internal combustion engine vehicles and battery electric vehicles (BEVs) [1–4]. First, hydrogen fuel cells are eco-friendly; they help dramatically reduce greenhouse gas emissions, as only water and heat are generated through the reaction of hydrogen and oxygen. Second, compared with the charging time of BEVs, that of FCEVs is short, and their driving distances are long, so they are suitable for long-distance transportation. Finally, FCEVs have a long lifespan because their performance degradation remains small despite an increase in operating time.

However, FCEVs use hydrogen gas as fuel, so various components are required [5,6]. Among them, the hydrogen valve performs the supplying and blocking functions of hydrogen gas, which is very important in the efficiency and safety of FCEVs [7,8]. In addition, the plunger magnetized by the electric force of the solenoid coil performs the opening and closing functions of the valve while reciprocating motion. Friction with the core occurs because of abnormal plunger movement during hydrogen valve operation. As a result, wear damage on the plunger surface is a factor that reduces the durability of the hydrogen valve. In particular, the degradation of mechanical characteristics and wear damage are further accelerated by hydrogen permeation in a hydrogen environment [9]. Therefore, surface treatment to improve hydrogen permeation resistance and mechanical characteristics is required.

Hydrogen permeation occurs in a stepwise process of surface adsorption, migration, diffusion, and filling [10]. First, the surface adsorption of hydrogen is caused by physical energy, such as pressure, and is mainly found at defects. The adsorbed hydrogen migrates and diffuses through the defects or grain boundaries inside the metal, trapping and filling certain areas. At this time, the partial pressure of hydrogen increases, which weakens the bonding force of the crystal or grain and, in severe cases, causes damage such as cracks, which significantly degrades the mechanical characteristics of the metal [11].

To investigate this problem, many researchers have used various experimental methods to permeate hydrogen. The most commonly used methods are electrochemical hydrogen charging method and high-pressure hydrogen gas testing method (ISO 15105 solid-gas testing method) [12,13]. Electrochemical hydrogen charging method, also known as cathodic hydrogen charging method, involves applying a cathodic DC current to the specimen to generate hydrogen at the surface. It is a relatively easily accessible method and exhibits high reproducibility through precise control of current density. In contrast, the high-pressure hydrogen gas method performs experiments under high pressure, which can easily simulate the real conditions. However, it requires a high-pressure hydrogen vessel, which brings with it safety concerns [14]. In addition, the electrochemical hydrogen charging methods can be used to investigate the accelerated damage and durability degradation of materials due to hydrogen permeation. Therefore, many researchers have adopted electrochemical hydrogen charging method to permeate the hydrogen [15–17].

The mechanical characteristics of hydrogen-permeated specimens can be evaluated in various ways. The most commonly used methods include microstructural analysis of metals, mechanical characteristics evaluation (indentation, scratch, friction/wear, slow strain rate test, etc.), and hydrogen gas adsorption and desorption analysis [18–20]. These methods are very effective in quantitatively or qualitatively evaluating the hydrogen permeation resistance of a material.

Using these methods, many researchers have investigated the hydrogen permeation resistance of various materials and coated specimens. K. S. D. Assis et al. investigated the damage of metals by hydrogen permeation and hydrogen embrittlement using cathodic hydrogen charging method and slow strain rate test, and found that these methods are very effective for evaluating hydrogen permeability [21]. In addition, C. M. Lepienski et al. investigated hydrogen embrittlement by electrochemical hydrogen charging method and evaluated the degree of hydrogen embrittlement by indentation and scratch experiments [22]. As a result, they reported that the hardness and adhesion strength of hydrogen-permeated specimens decreased, which was attributed to crack formation and decreasing in the elastic force due to hydrogen embrittlement. Tamura et al. investigated the hydrogen permeation resistance of specimens coated with Al_2_O_3_, TiN, and TiC, and found that the Al_2_O_3_ coating layer with a small grain size exhibited the best hydrogen permeation resistance [23]. In addition, Daub et al. applied CrN, TiAlN, and TiCrN coatings to Zr-4 tubes to reduce hydrogen permeability [24]. This was attributed to the excellent adhesion strength and free-defects of CrN and TiAlN coatings.

Electroless nickel plating has also been investigated to improve the hydrogen permeation resistance. Electroless nickel plating has various advantages over electrolytic nickel plating as a surface treatment technology [25]. Regardless of the substrate shape, a uniform nickel plating layer with a relatively strong adhesion strength is formed [26]. As the nickel plating layer is chemically stable, has excellent corrosion resistance, and has high wear resistance because of its lubricating characteristics, it is applied in various industrial fields [27,28]. In particular, nickel has a very low hydrogen diffusion coefficient of about 5 × 10^−11^ m^2^/s [29]. Hillier et al. showed that the nickel plating layer acts as a barrier to hydrogen permeation because of the low hydrogen diffusion coefficient of nickel [30]. Furthermore, Samanta et al. found that the electroless nickel plating layer exhibits lower hydrogen-induced embrittlement than the electrolytic nickel plating layer does [31]. This may be attributed to the excellent hydrogen barrier characteristics of the electroless nickel plating layer. However, Hino et al. reported that hydrogen permeation affects the electroless nickel plating layer more than it does the electrolytic nickel plating layer [32]. This is because the crystal structure of the electroless nickel plating layer and the pits on the plating surface affect hydrogen permeability.

Nevertheless, electroless nickel plating can be mass-produced at a low cost, so it is highly effective in reducing costs in the hydrogen industry [33,34]. Complex considerations of hydrogen permeation resistance and mechanical characteristics are required, for the application of electroless nickel plating to the hydrogen industry. In particular, there is a need to investigate the application of hydrogen valve plungers in which friction and wear occur simultaneously in a hydrogen environment.

In this research, electroless nickel plating was performed on free-cutting steel used with the plunger of a hydrogen valve. The effects of hydrogen on the mechanical characteristics of electroless nickel plating were investigated through hydrogen permeation, indentation, and scratch and sliding wear experiments of the electroless nickel plating samples.

## 2. Experimental method

### 2.1 Preparation of sample and electroless nickel plating process

The substrate used in this research is free-cutting steel, and its chemical composition is shown in Table 1. Before the electroless nickel plating, the substrate was cut to a size of 20 mm × 20 mm × 5 mm and surface polished to SiC abrasive paper grit # 1200. The polished sample was cleaned with ultrasonic waves using acetone and distilled water and then dried in a vacuum dryer for 24 h. A nickel plating layer was then deposited on the surface of the free-cutting steel using the electroless nickel plating method, and the detailed process is presented in Table 2. Energy dispersive spectroscopy (EDS, AZTec Energy, Oxford Instruments, Abingdon, UK) analysis was also performed to measure the phosphorus content of the electroless nickel plating layer.

**Table 1:** Chemical composition of free-cutting steel (SUM 24L).

C	Mn	P	S	Pb	Fe
0.05	0.97	0.057	0.294	0.26	Bal.

**Table 2:** Chemical composition and parameter of electroless nickel plating bath.

Chemical	Quantity(g/L)	Parameter	Value
Nickel sulphate	20–30	pH of solution	4.5–5.0
Sodium hypophosphite	25–30	Deposition temp. (°C)	85
Lactic acid	8–10	Time (min)	20
Sodium acetate hydrate	15–20	Expected phosphorus content (wt%)	8–10

### 2.2 Characteristics of electroless nickel plating layer

Crystal analysis of the nickel plating layer was performed using an X-ray diffraction (XRD, D Max-2500/PC, Rigaku, Tokyo, Japan) analyzer. Cu K_α_ was used as the measurement condition; the step size was 0.02°, the scan time was 1.0 s/step, and 2-theta degree was measured in the range of 30° ~ 90°. Cross-section observation and chemical composition analysis of electroless nickel-plated free-cutting steel were also performed using a field emission scanning electron microscope (FE-SEM, JSM-6700F, JEOL, Tokyo, Japan) and EDS.

### 2.3 Hydrogen permeation experiment

**Fig 1:**
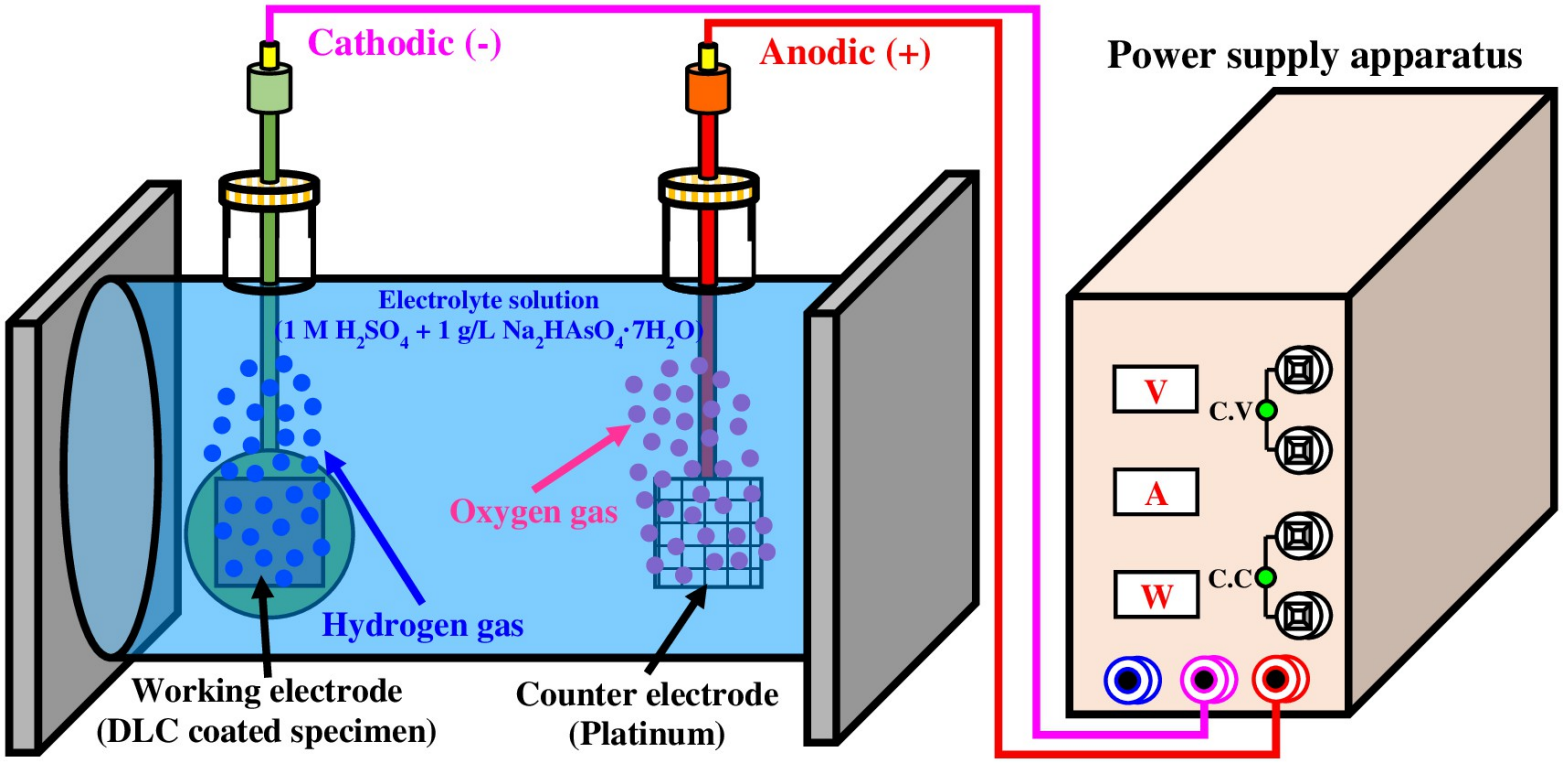
Schematic of hydrogen permeation experiment with cathodic hydrogen charging method in 1 M H_2_SO_4_ + 1 g/L Na_2_HAsO_4_·7H_2_O solution.

In this research, we investigated the damage and durability degradation of electroless nickel plating layers due to hydrogen permeation as an accelerated lab-scale experiment before simulating the real environment. To investigate the hydrogen permeation resistance of electroless nickel-plated free-cutting steel, hydrogen was artificially charged in a 1 M H_2_SO_4_ + 1 g/L Na_2_HAsO_4_·7H_2_O solution at an applied current density of 10 mA/cm^2^ for 0, 6, 12, and 24 h. The cathodic hydrogen charging method was adopted for hydrogen charging, and the specimen and platinum electrode were configured as the cathode and anode, respectively. In addition, a stable DC current was applied using a power supply apparatus (E3642A, KEYSIGHT TECHNOLOGIES, Seoul, Republic of Korea). Details of the hydrogen permeation experiment are shown in the schematic in Fig 1. The cathodic hydrogen charging method is a popular method for investigation of the electrochemical hydrogen embrittlement and has been utilized by many researchers because it does not require high pressure hydrogen gas and a corresponding vessel [35–37]. In addition, arsenic in an acidic solution prevents hydrogen from molecularizing, allowing hydrogen to be charged into the specimen as ions or atoms [38]. To analyze the hydrogen permeated specimen, the surface roughness of the sample was then measured using a three-dimensional (3D) confocal laser microscope (OLS-5000, OLYMPUS, Tokyo, Japan), and the effects of hydrogen permeation on the electroless nickel plating layer were observed with FE-SEM.

### 2.4 Micro indentation experiment

For the analysis of mechanical characteristics, a micro-indentation tester (MCT3, Anton paar, Graz, Austria) equipped with a 68° diamond Vickers indenter was used. Considering the thickness of the electroless nickel plating layer, the maximum load was set to 100 mN so that the maximum displacement of the indenter was less than 1 μm. The loading rate and the holding time at the maximum load were 100 mN/min and 5 s, respectively. The maximum load of 100 mN for the indentation experiment was chosen to minimize the influence of the base material, and the maximum indentation depth was within 800 nm (20% of the coating thickness), taking into account the average thickness of electroless nickel-plated layer of 4 μm. In addition, the Poisson’s ratio of the sample was set to 0.27 (SUM 24L) and 0.31 (Electroless nickel plating), respectively. The Vickers hardness was calculated with the Oliver and Pharr method using the load—displacement curve obtained after the micro-indentation experiment [39]. As the surface roughness is non-uniform because of electroless nickel plating characteristics, indentation experiments were repeated 25 times under the same conditions for reproducibility, and then among the remaining data, excluding the maximum and minimum values, data close to the average were used.

### 2.5 Micro scratch experiment

To measure the adhesion strength of the electroless nickel plating layer based on hydrogen permeation, a micro-scratch tester (MCT3, Anton paar, Graz, Austria) equipped with an optical microscope, an acoustic emission detection system, a tangential friction force sensor, and a permeation depth measurement sensor were used. In addition, a scratch experiment was performed using a diamond Rockwell C-type indenter with an angle of 120° and a diameter of 100 μm. The scratch distance was 2 mm, the load increased linearly from 0.02 N to 20 N, and the scratch and load rates were set at 1 mm/min and 10 N/min, respectively. The adhesion strength of the electroless nickel plating layer was analyzed using the acoustic emission signal obtained after the scratch experiment and the optical microscopy. To ensure reproducibility, the scratch experiment was repeated seven times under the same conditions, and scratch data with adhesion values close to the average were used.

### 2.6 Sliding wear experiment

A tribometer was used for the sliding wear experiments, and a ball-on-disk type was adopted as the test method. The counter balls used in the experiment were alumina balls with a diameter of 6 mm and a Vickers hardness of 1,650 HV. As experimental conditions, the rotation radius, speed, applied load, and sliding distance of the disk were set to 3 mm, 3 cm/s, 5 N, and 200 m, respectively. The temperature and humidity were maintained at 25°C and 50%, respectively. After the sliding wear experiments, the friction coefficients were compared based on the sliding distance, the damage to the wear track was measured, and the surface of the worn sample was observed using a 3D confocal laser microscope.

## 3. Result and discussion

**Fig 2:**
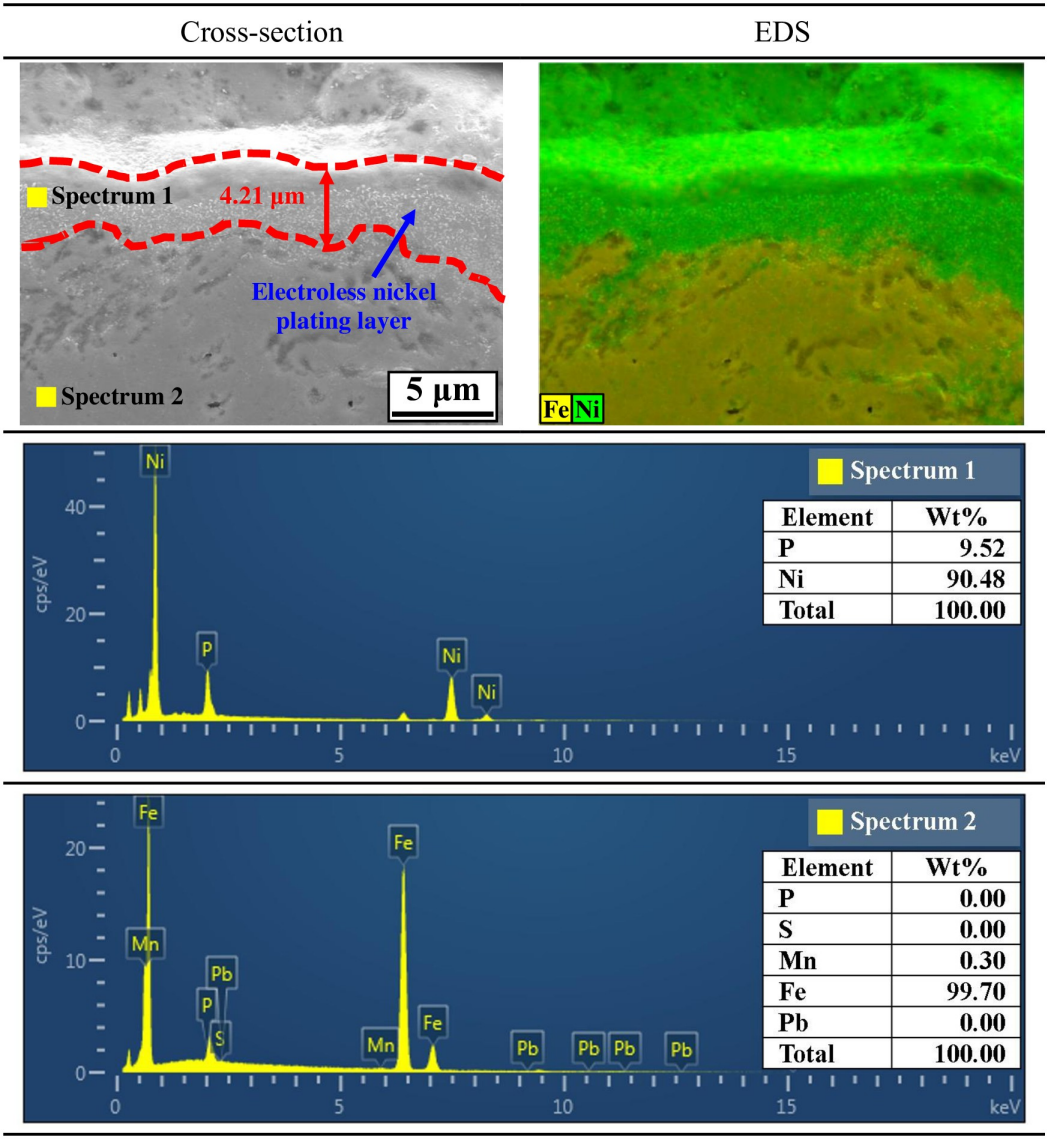
Cross-sectional images and EDS analysis before hydrogen permeation experiment of electroless nickel-plated free-cutting steel.

Fig 2 presents the cross-sectional observational results of electroless nickel-plated free-cutting steel using FE-SEM and EDS. As a result of cross-sectional observation, the electroless nickel plating layer was densely deposited. In addition, the thickness ranged from about 2.81 μm to 5.61 μm, and the average thickness was about 4.21 μm. No defects were observed at the interface between the nickel plating layer and the substrate, indicating strong deposition. Chemical composition mapping by EDS demonstrates that iron was predominantly measured for the substrate, whereas nickel was predominantly measured for the nickel plating layer. In addition, the component analysis of the nickel plating layer indicates that a nickel—phosphorus alloy was deposited with 90.48 wt% and 9.52 wt% nickel and phosphorus, respectively. In the electroless nickel plating in this research, a nickel—phosphorus alloy was thus formed within the phosphorus content range.

**Fig 3:**
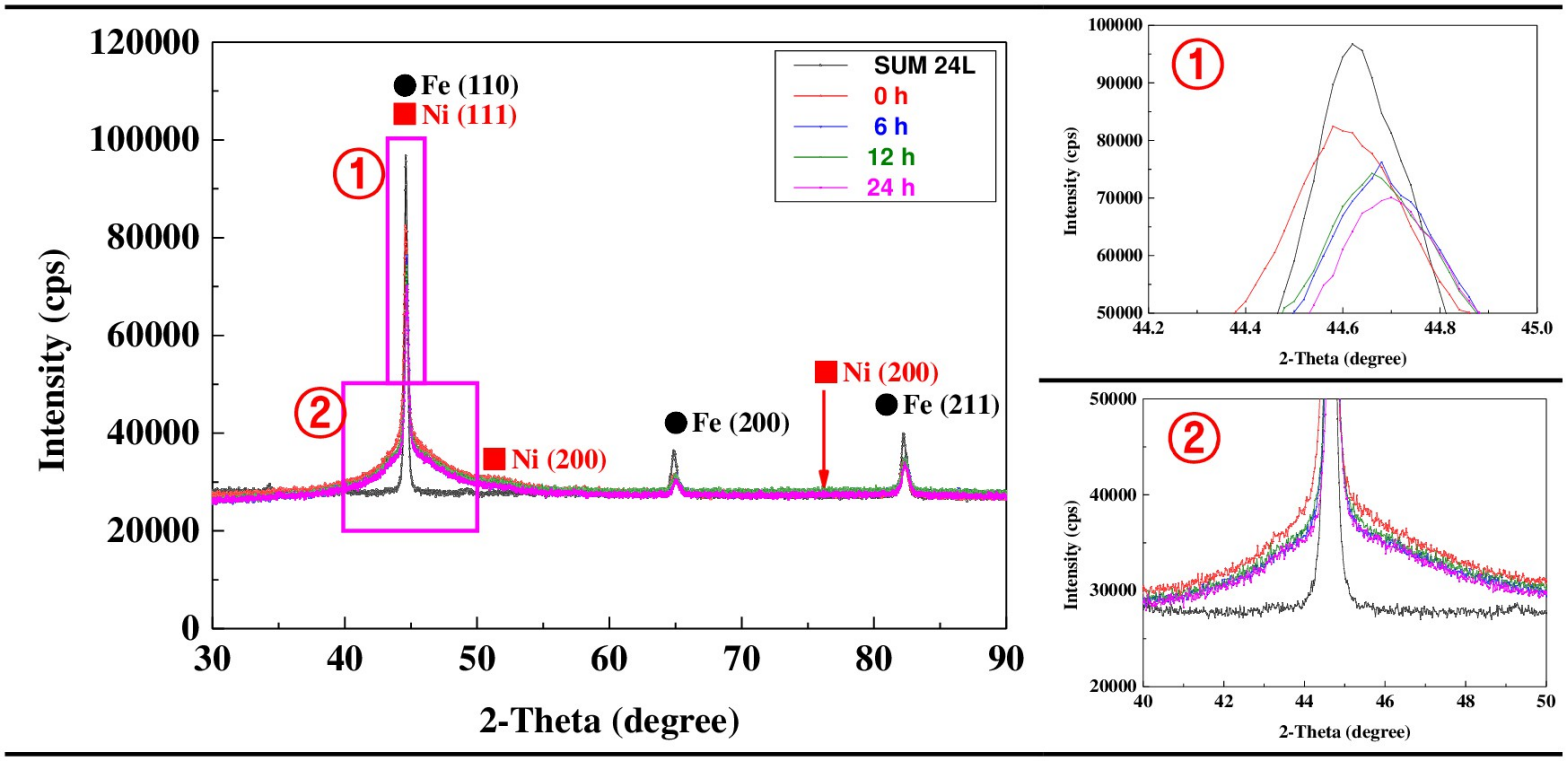
XRD analysis after hydrogen permeation experiment of electroless nickel-plated free-cutting steel.

Fig 3 presents the XRD analysis results of the electroless nickel plating layer and the free-cutting steel in terms of hydrogen permeation time. In the case of free-cutting steel, strong peaks appeared at 44.62°, 64.86°, and 82.26°, which are the crystal planes of iron corresponding to (110), (200), and (211), respectively. The electroless nickel-plated sample exhibited a relatively low peak at the 2-theta degree, which was almost similar to that of the free-cutting steel. In general, when the phosphorus content is low during electroless nickel plating, a nickel plating layer with a nanocrystalline structure is deposited [40]. This nickel crystalline structure displays a face-centered cubic phase [41]. Therefore, the nickel crystalline structure presents peaks at 2-theta degrees of 44° (111), 51° (200), and 76° (220) in the XRD analysis [42]. However, as the phosphorus content increases, nickel—phosphorus alloys are deposited and exhibit an amorphous structure [43]. The electroless nickel plating layer in this research contained about 9.52% phosphorus. Therefore, it is thought that the amorphous nickel—phosphorus alloy was predominantly deposited on the substrate surface, resulting in a strong peak at the 2-theta degree corresponding to free-cutting steel. In particular, the relative decrease in and the broadening of the peak at 44.62° are thought to be results of the nanocrystalline nickel structure and the amorphous nickel—phosphorus alloy structure being deposited together during electroless nickel plating [44,45].

**Fig 4:**
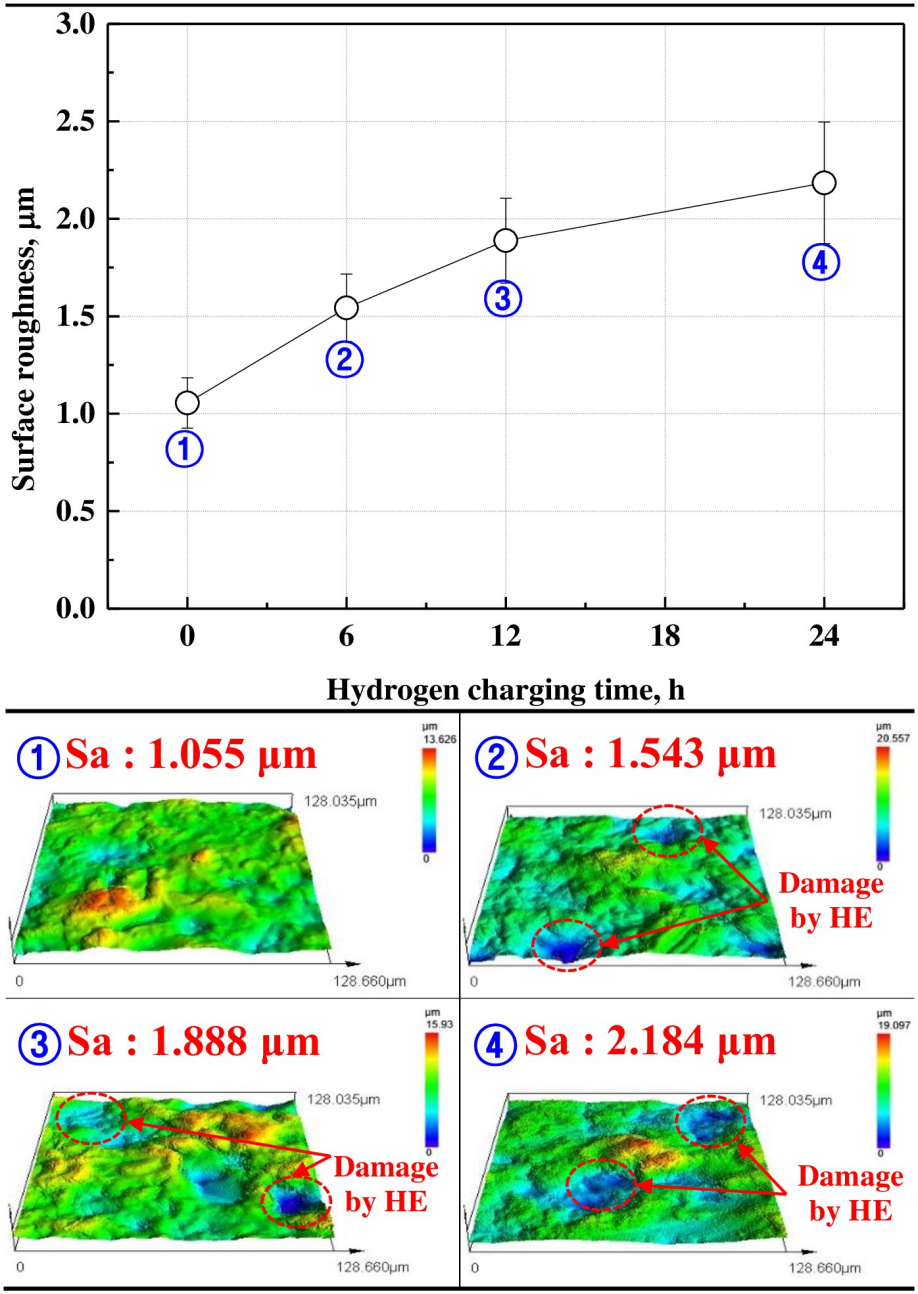
Surface roughness after hydrogen permeation experiment of electroless nickel-plated free-cutting steel.

Fig 4 exhibits the results of the surface observations and surface roughness measurements of the hydrogen-permeated electroless nickel plating layer with a 3D confocal laser microscope. Surface roughness was measured over 7 points to ensure data reproducibility, and the data that approximated to the average and standard deviation were plotted excluding the maximum and minimum. The surface of the electroless nickel plating layer was damaged due to hydrogen permeation. In addition, surface damage and roughness increased as the hydrogen permeation time increased. In particular, the surface roughness before hydrogen permeation was 1.055 μm, and after hydrogen permeation for 24 h, it increased approximately twice to a maximum of 2.184 μm. In general, hydrogen solubility and the hydrogen diffusion coefficient are very important factors as plating characteristics to prevent hydrogen permeation [46]. In the case of electroless nickel plating, the hydrogen diffusion coefficient is very low because of the inherent characteristics of nickel [29]. However, as depicted in the XRD analysis in Fig 3, electroless nickel plating has relatively high hydrogen solubility because of the formation of a nanocrystalline nickel structure and an amorphous nickel—phosphorus alloy [47]. Therefore, hydrogen ions or atoms are easily trapped inside the electroless nickel plating layer, causing plastic deformation according to the dislocation movement of the crystal [48]. This process is assumed to continue in a specific area of the surface, thus damaging the surface of the nickel plating layer and increasing surface roughness.

**Fig 5:**
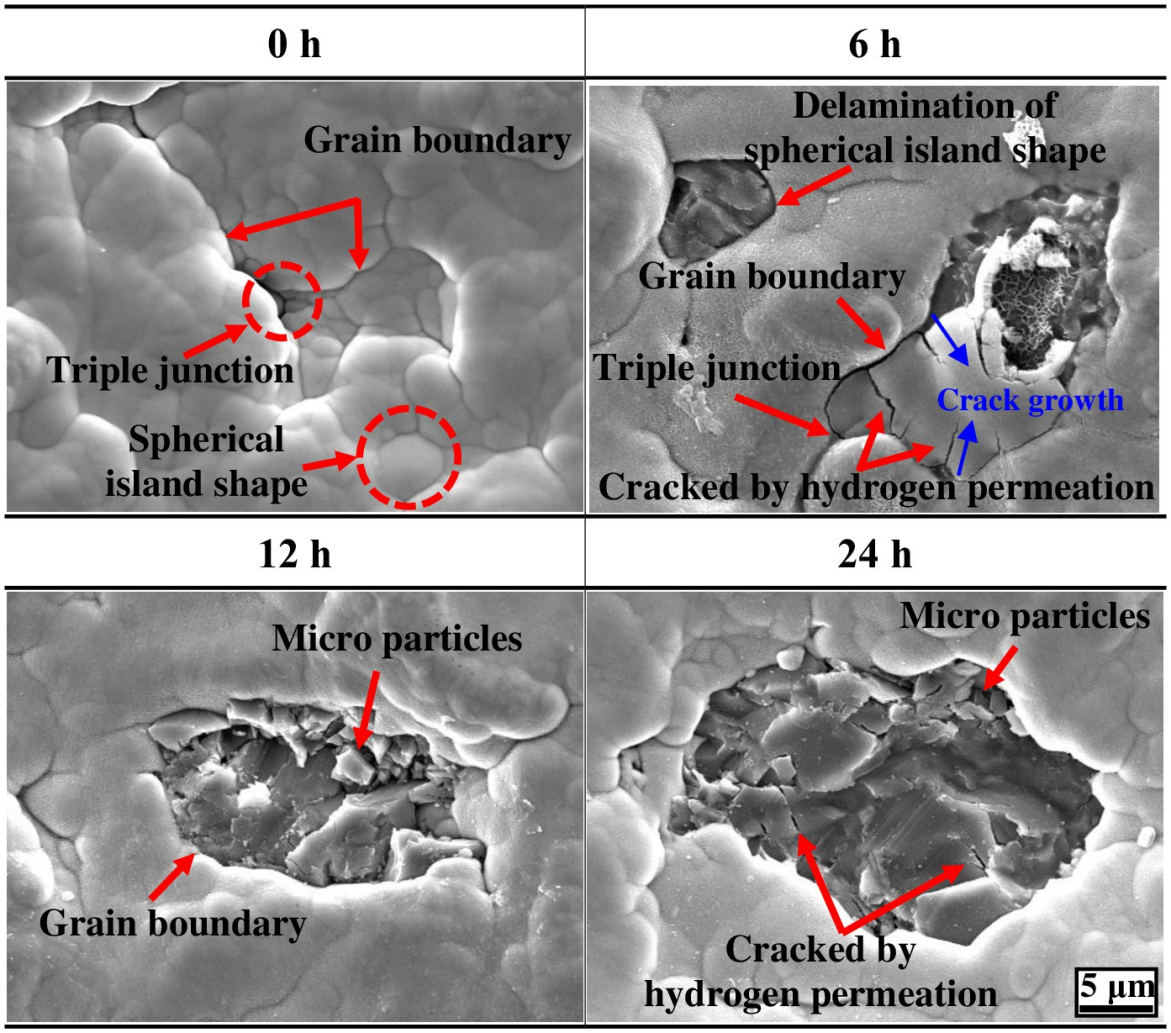
Surface morphologies after hydrogen permeation experiment of electroless nickel-plated free-cutting steel.

Fig 5 depicts the surface observational results of the electroless nickel plating layer with FE-SEM. In general, electroless nickel plating exhibits an island-like growth mode depending on the plating mechanism [49]. In particular, when the content of sodium hypophosphite (NaH_2_PO_2_), which plays the role of the reducing agent, increases, the grain size increases, and the surface smoothens [50]. Micro-defects, such as pinholes and pores, are also reduced, resulting in increased wear resistance [51–53]. This trend was also observed in this research. The surface of the electroless nickel-plated sample, which is not hydrogen permeated, grew in the form of large spherical islands with a micro-grain structure. Also, micro-defects such as pinholes and pores were not observed. Micro-defects, such as pinholes and pores, were not observed. In general, hydrogen ions or atoms permeate the inside of the crystalline structure through micro-defects formed on the surface of metal or plating layers [54]. Therefore, electroless nickel plating without defects on the surface is effective in preventing hydrogen permeation. Because of the characteristics of electroless nickel plating, however, grain boundaries and triple junctions are formed between the micro-grains while they grow into spherical island-like shapes [55]. As these grain boundaries and triple junctions act as passages through which hydrogen ions or atoms can permeate the nickel plating layer, they are considered factors that deteriorate the mechanical characteristics and durability of the nickel plating layer and substrate. In particular, triple junctions are known as microstructural defects with inherent metal physical/chemical characteristics, and their durability depends on the crystal size [56–58]. That is, as the crystal size increases, the volume fraction of the triple junction with respect to the volume fraction of the total boundary region is small, and the effects are negligible. However, as the crystal size is small, the volume fraction of the triple junction increases, acting as a defect that cannot be ignored. This can be seen in the observation of the surface damage shape of the electroless nickel plating after hydrogen permeation. In the case of hydrogen permeation for 6 h, damage to the electroless nickel plating layer was clearly observed. The nickel plating layer was peeled off as an island-shaped mass composed of micro-grains, and the edge of the damaged area appeared in a curved shape. Cracks in the nickel plating layer were found in the grain boundary region and the triple junction between crystals. In particular, cracks were observed more significantly in the grain boundary region than in the triple junction. As the crystal size of the electroless nickel plating layer is relatively large, the grain boundary region is considered the main defect area for hydrogen permeation rather than the triple junction. In addition, the cracks grew from grain boundary toward the intergranular. This is because the grain boundaries and triple junctions are hydrogen permeation pathways and increasing area of the hydrogen partial pressure. As the hydrogen permeation time increased, the size of the surface damage further increased. Micro-cracks and micro-particles were clearly observed inside the damaged area. These micro-particles are assumed to increase the wear rate by acting as debris (third bodies) during the friction and wear experiment. In addition, the nickel plating layer (0 h sample) before hydrogen permeation has compressive residual stress in the crystal structure [59,60]. This compressive residual stress increases the hardness by delaying the plastic deformation of the metal. However, the compressive residual stress of the sample in which local delamination of the nickel plating layer occurred after hydrogen permeation for 12 h was reduced because of damage in and thickness reduction of the nickel plating layer [61,62]. Eventually, the mechanical characteristics deteriorated as the compressive residual stress decreased.

**Fig 6:**
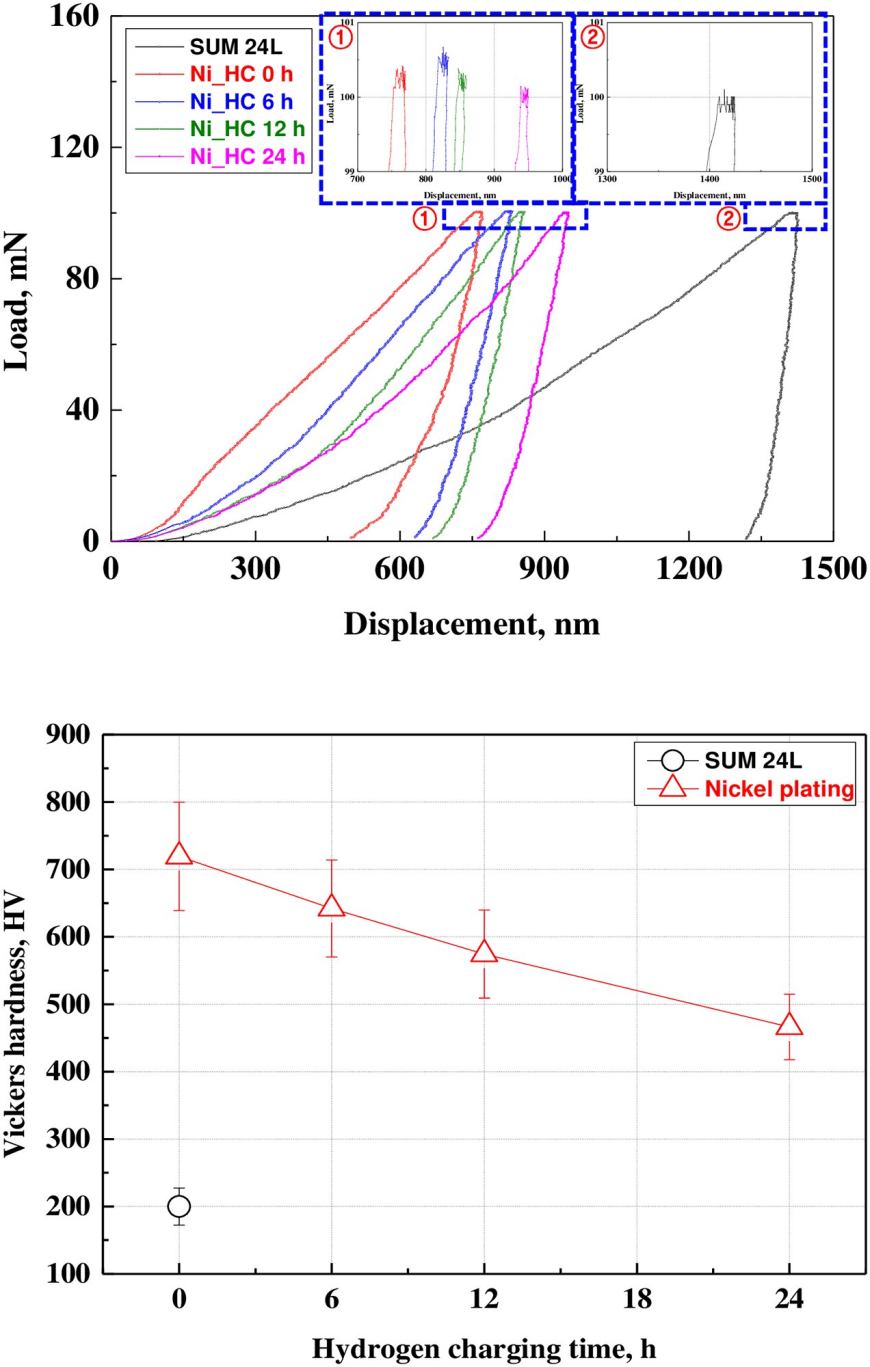
Load-displacement curve and Vickers-hardness after hydrogen permeation experiment of electroless nickel-plated free-cutting steel. (a) Load-displacement curve, (b) Vickers-hardness.

Fig 6 presents the results of the indentation experiment of the free-cutting steel and electroless nickel plating layer. The load was held for 5 s at the maximum load of 100 mN, and the analysis of creep behavior depicts that all samples presented creep behavior in which indenter displacement increased. In the case of free-cutting steel, creep behavior was observed more clearly. The creep behavior of the electroless nickel plating with hydrogen permeation time did not depict a significant difference. In general, when the plating layer was damaged and the thickness was reduced, creep behavior was affected by the substrate under the same load conditions. In the case of the electroless nickel plating layer, the surface was damaged by hydrogen permeation. However, the thickness of the electroless nickel plating layer was almost constant and was not affected by the substrate, so the creep behavior was considered similar to the increase in hydrogen permeation time. However, the Vickers hardness measurement demonstrates that the hardness of electroless nickel plating decreased from 719 HV to a maximum of 466 HV as the hydrogen permeation time increased. This is attributed to surface damage as a result of hydrogen permeation and to the formation of voids in the nickel plating layer, which reduces the bonding strength and compressive residual stress of the plating layer, resulting in a decrease in hardness [63,64]. However, all electroless nickel plating samples exhibited significantly higher hardness than 199 HV—the Vickers hardness of the free-cutting steel used as the substrate—and an increase of up to three times or more. Therefore, electroless nickel plating improved mechanical characteristics by improving the Vickers hardness of the free-cutting steel. The creep characteristics of the electroless nickel plating layer as a result of hydrogen permeation did not change, and the decrease in hardness as a result of surface damage was thought to affect mechanical characteristics and durability.

**Fig 7:**
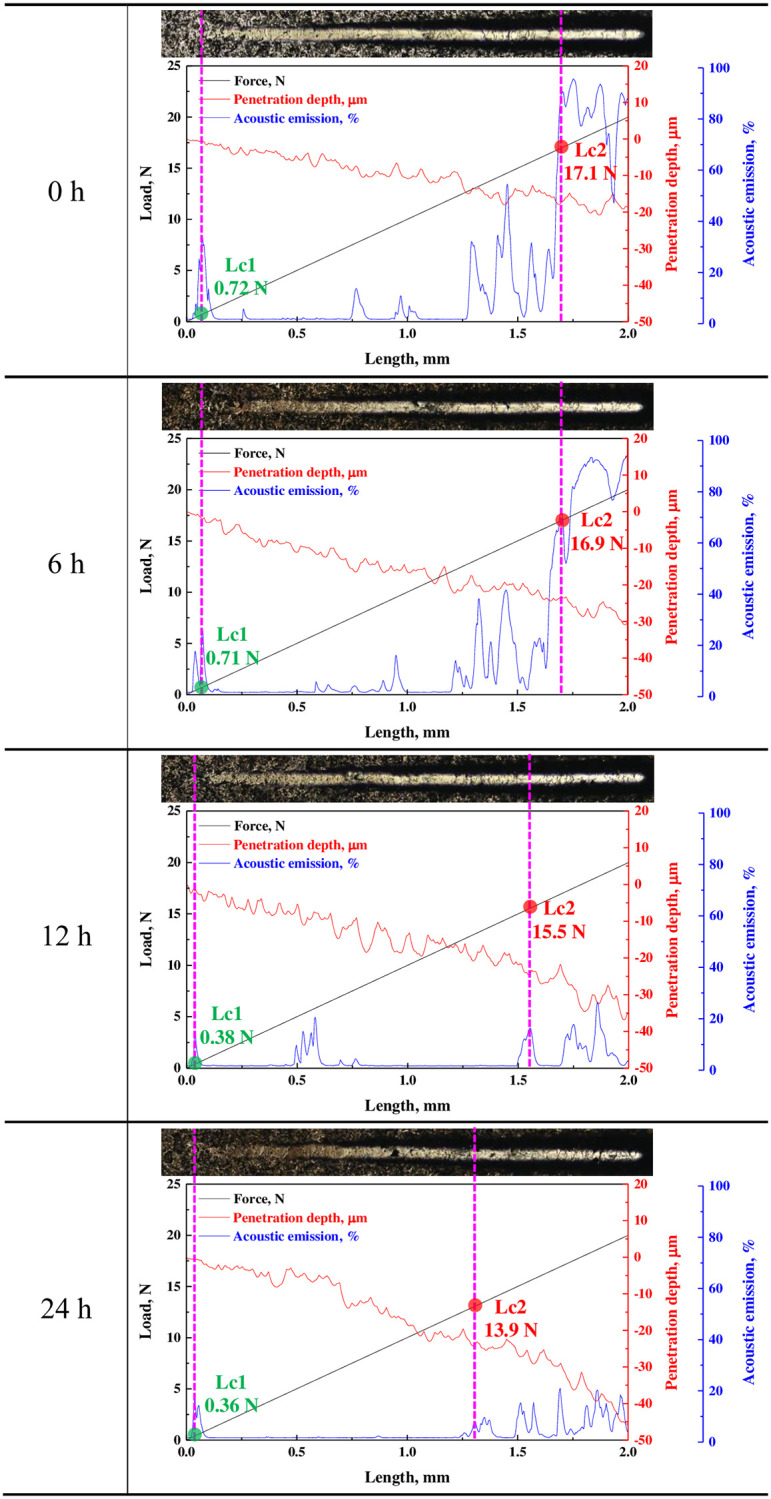
Scratch experiment results after hydrogen permeation experiment of electroless nickel-plated free-cutting steel.

Fig 7 exhibits the results of the scratch experiment to analyze the adhesion strength of the electroless nickel plating layer by hydrogen permeation. Before hydrogen permeation, the initial peak of the acoustic emission signal corresponding to the crack in the nickel plating layer was measured at a load of about 0.72 N, which was indicated as critical load 1 (Lc1). However, as the hydrogen permeation time increased, the critical load corresponding to the initial peak of the acoustic emission signal decreased. In the case of the hydrogen permeation experiments for 0 h and 6 h, critical load 2 (Lc2), which corresponds to the adhesion strength of the electroless nickel plating layer, had values of 17.1 N and 16.9 N, respectively, indicating very high acoustic emission signal values. However, the adhesion strength decreased to 15.5 N and 13.9 N in the hydrogen permeation experiments for 12 h and 24 h, respectively. The acoustic emission signal value also rapidly decreased. In general, the acoustic emission signal value presented in the scratch experiment included critical load 1 (Lc1), which appeared when the plating layer cracked. Critical load 2 (Lc2) was measured when the plating layer was peeled off from the substrate by complete fracture of the plating layer [65,66]. With these parameters, the adhesion strength between the plating layer and the substrate was evaluated. That is, as the bonding and adhesion strengths of the plating layer were strong, cracks and fractures appeared more distinctly according to the load of the indenter; as a result, the acoustic emission signal value appeared large. Therefore, when the hydrogen permeation time was short, the bonding and adhesion strengths of the electroless nickel plating layer were relatively high, so the acoustic emission signal value was large.

**Fig 8:**
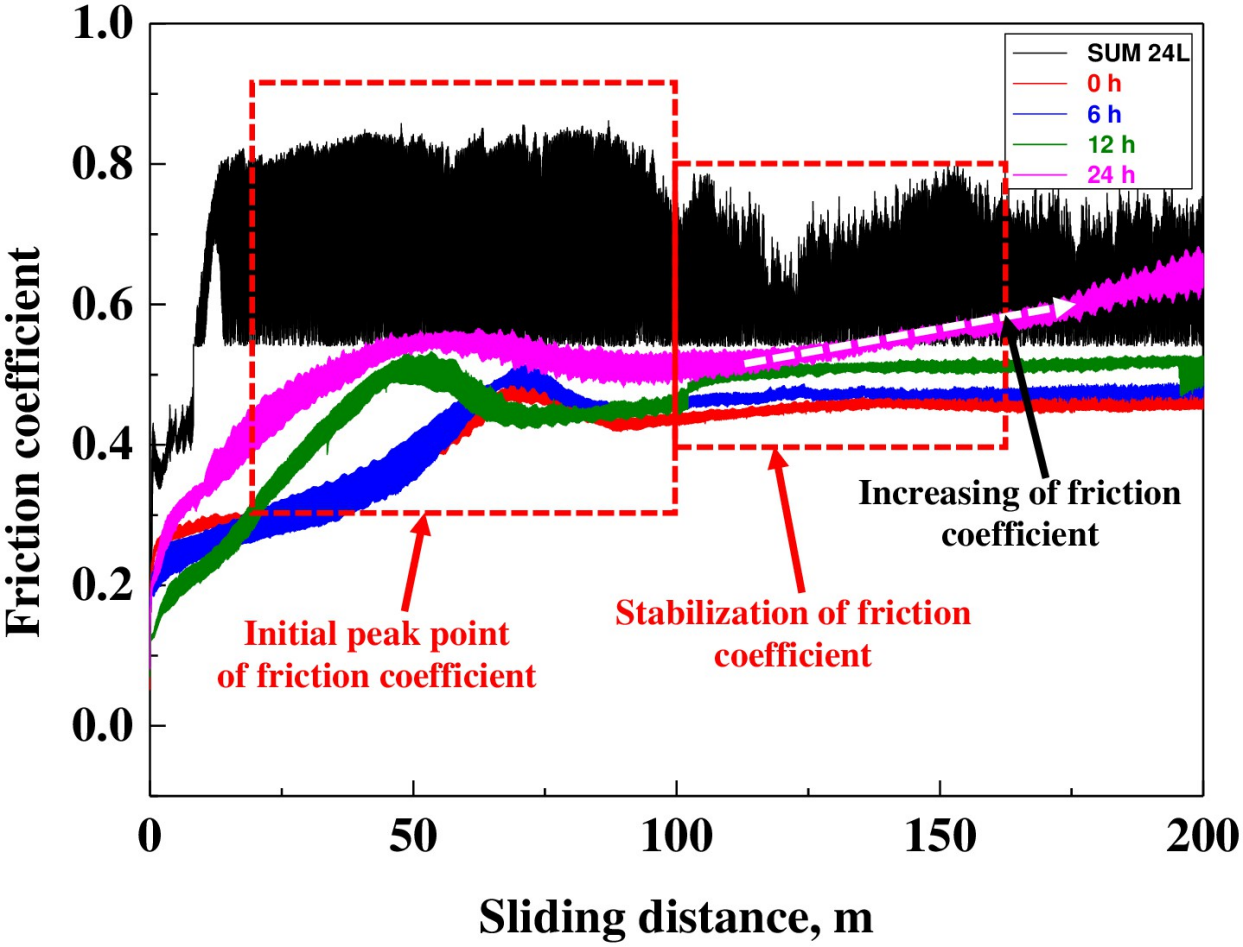
Friction coefficient curves after hydrogen permeation experiment of electroless nickel-plated free-cutting steel.

Fig 8 presents the results of the sliding wear experiment after hydrogen permeation to analyze the effects of hydrogen on the electroless nickel plating layer. The comparison result of friction coefficients based on the sliding distance demonstrates that the friction coefficient stabilized after the initial peak value appeared in all experimental conditions. It is known that the initial peak value in the friction coefficient curve appears because of the deformation of the counter ball and the increase in the number of wear debris on the surface of the sample [67,68]. The results indicate that the sliding distance corresponding to the initial peak value of the friction coefficient demonstrated the lowest value of about 13 m for free-cutting steel. An initial peak value was observed at a sliding distance of about 73 m for the electroless nickel plating sample before hydrogen permeation, which was improved by about 5.6 times compared to that of free-cutting steel. However, as the hydrogen permeation time increased, the value decreased to about 45 m (24 h sample), indicating a deterioration in wear resistance of about 38%. In addition, the average friction coefficient of all samples in the stabilization period presented the highest value (0.64 μ) of free-cutting steel, which increased by a maximum of 16% from 0.45 μ (0 h) to 0.52 μ (24 h) with hydrogen permeation time. In general, the factors that affect the friction coefficient are very diverse, such as surface roughness, temperature, applied load, inherent metal characteristics (elasticity, hardness, etc.), and sliding speed [69]. Among them, the factors found to affect the friction coefficient in this research were the surface roughness and the characteristic variations of the sample by hydrogen permeation. As shown in Fig 4., the surface roughness of the electroless nickel plating layer increased as the hydrogen permeation time increased; as a result, the friction coefficient increased with wear during the sliding wear experiment. The hardness also decreased along with a decrease in the bonding strength of the nickel plating layer and the formation of cracks by local surface damage, which affected the friction coefficient. However, in the case of the electroless nickel plating layer with hydrogen permeation for 24 h, the friction coefficient was lower up to a sliding distance of 130 m compared with that of the free-cutting steel. Therefore, the electroless nickel plating layer exhibited better wear resistance.

**Fig 9:**
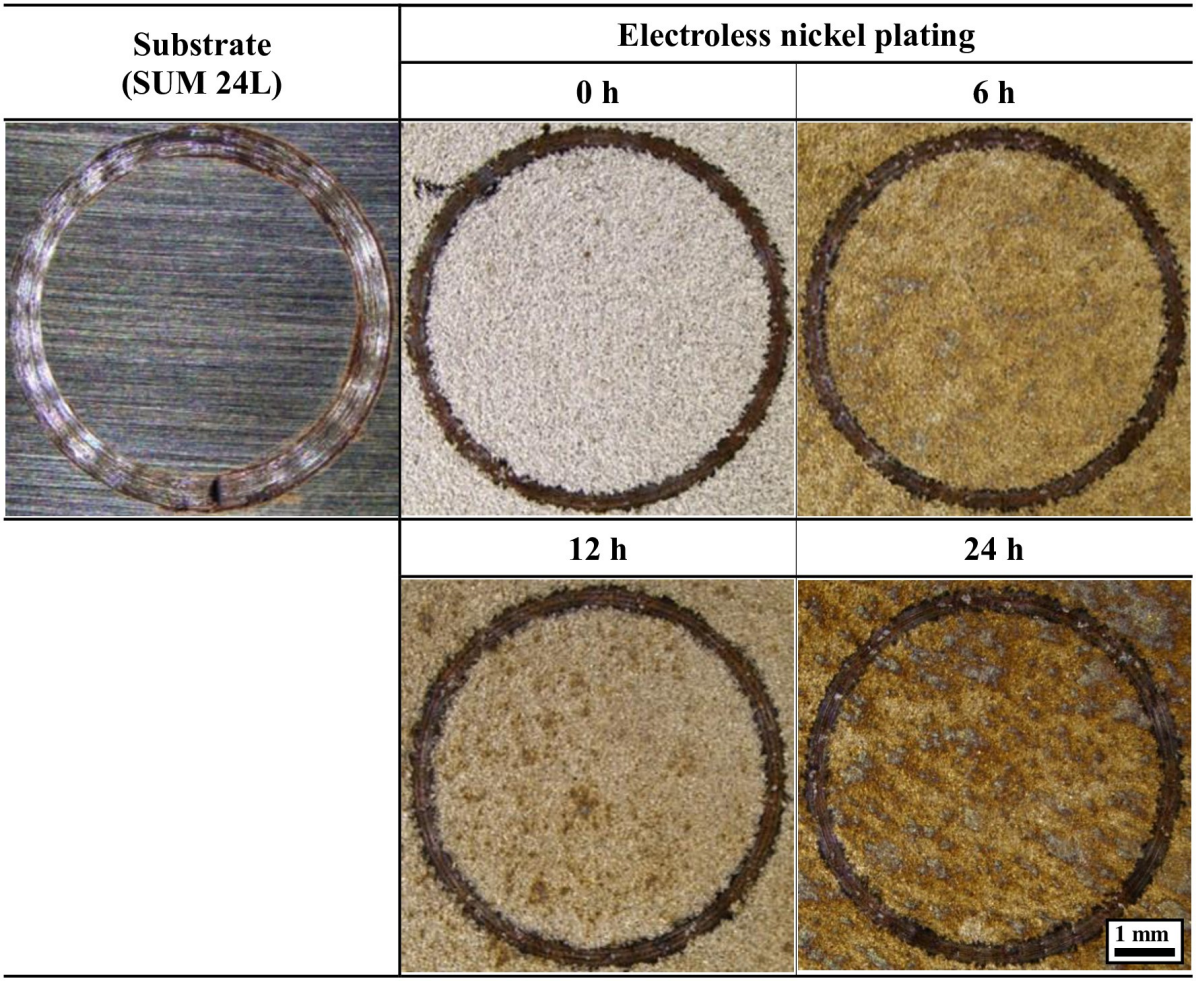
Wear-down image after sliding friction experiment of electroless nickel-plated free-cutting steel and substrate with/without hydrogen permeation.

Fig 9 depicts the results of observing the wear track of the electroless nickel plating layer with hydrogen permeation and that of the free-cutting steel after the sliding wear experiment using a stereo microscope. The wear tracks were clearly observed in all samples. The free-cutting steel presented a clear trend of abrasive wear, and a smooth, round shape was observed at the edge of the wear track. In the case of electroless nickel plating, the edge of the wear track was worn in a relatively rough shape. In particular, the number of white spots in the wear track increased as the hydrogen permeation time increased. These white spots appear when the substrate is exposed locally via adhesive wear [70]. It is considered that this adhesive wear trend increases as the bonding strength and adhesion strength of the electroless nickel plating layer decreases as a result of hydrogen permeation.

**Fig 10:**
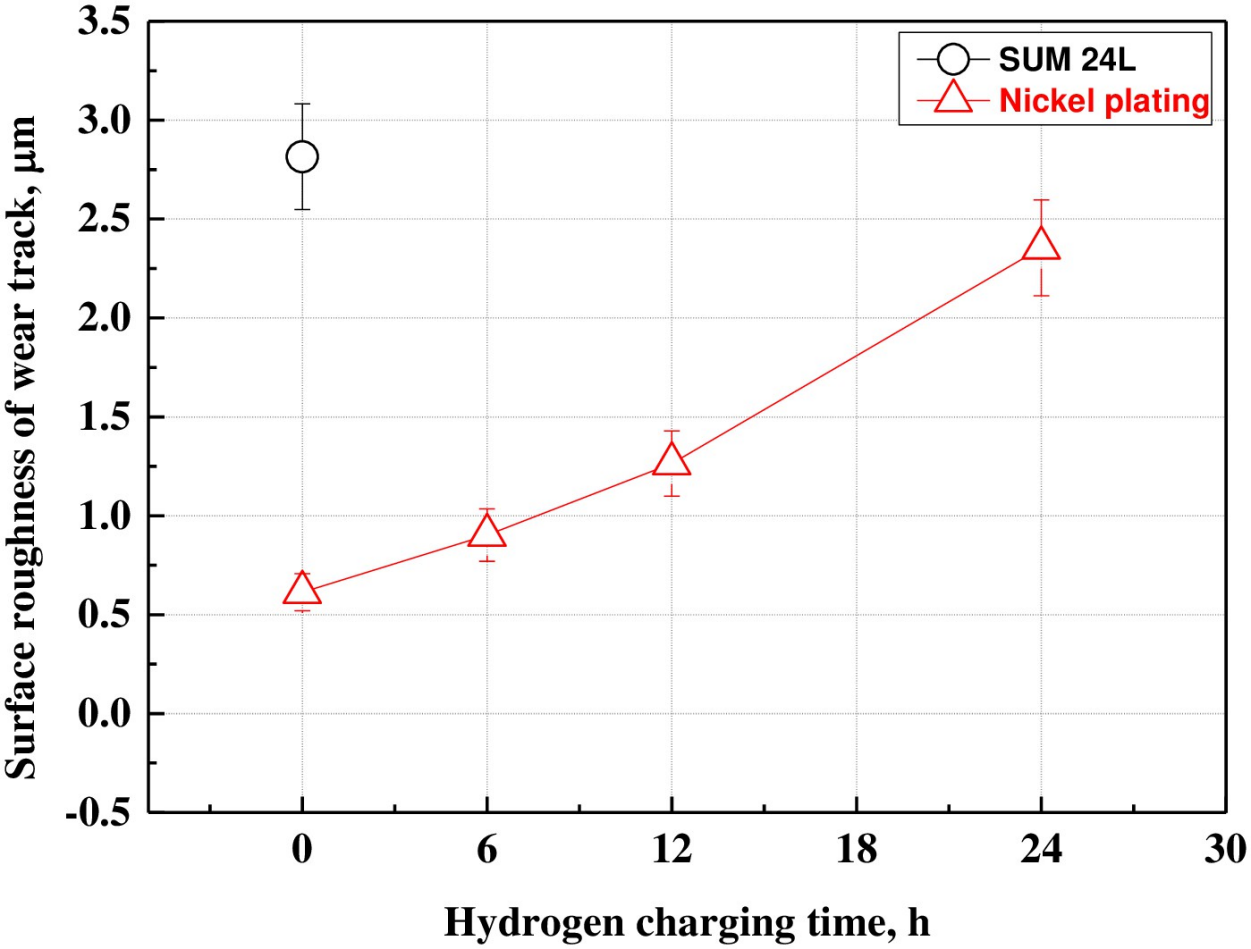
Surface roughness of wear track after sliding friction experiment of electroless nickel-plated free-cutting steel and substrate with/without hydrogen permeation.

Fig 10 compares the surface roughness(Sa) in the wear tracks of free-cutting steel and electroless nickel plating. In order to compare the surface roughness of the wear track, the surface roughness of the same area inside the wear track was measured as a target. In the case of free-cutting steel with relatively low hardness, the surface roughness was measured to be the largest at 2.815 μm due to abrasive wear of the round shape (counter ball-like shape). In the case of electroless nickel plating, abrasive wear of the flat shape and local adhesive wear appeared in combination. Therefore, the surface roughness increased by about 3.84 times from 0.613 μm (0 h sample) to a maximum of 2.354 μm (24 h sample) with an increase in the number and size of damaged parts by adhesive wear. Surface roughness was affected by wear depth and width, with wear depth exerting a greater effect.

**Fig 11:**
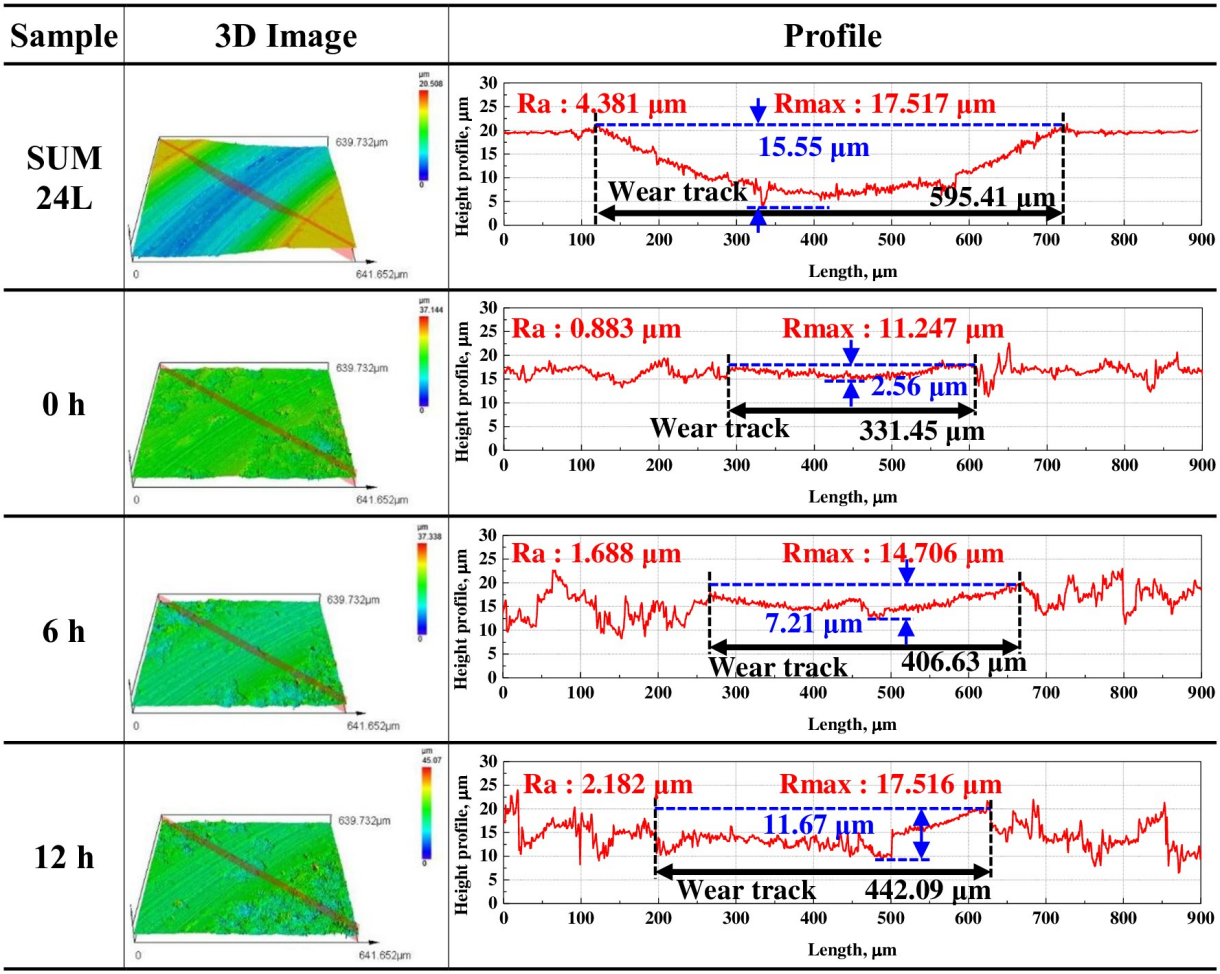
3D images and profile analysis of wear track after sliding friction experiment of electroless nickel-plated free-cutting steel and substrate with/without hydrogen permeation.

**Fig 12:**
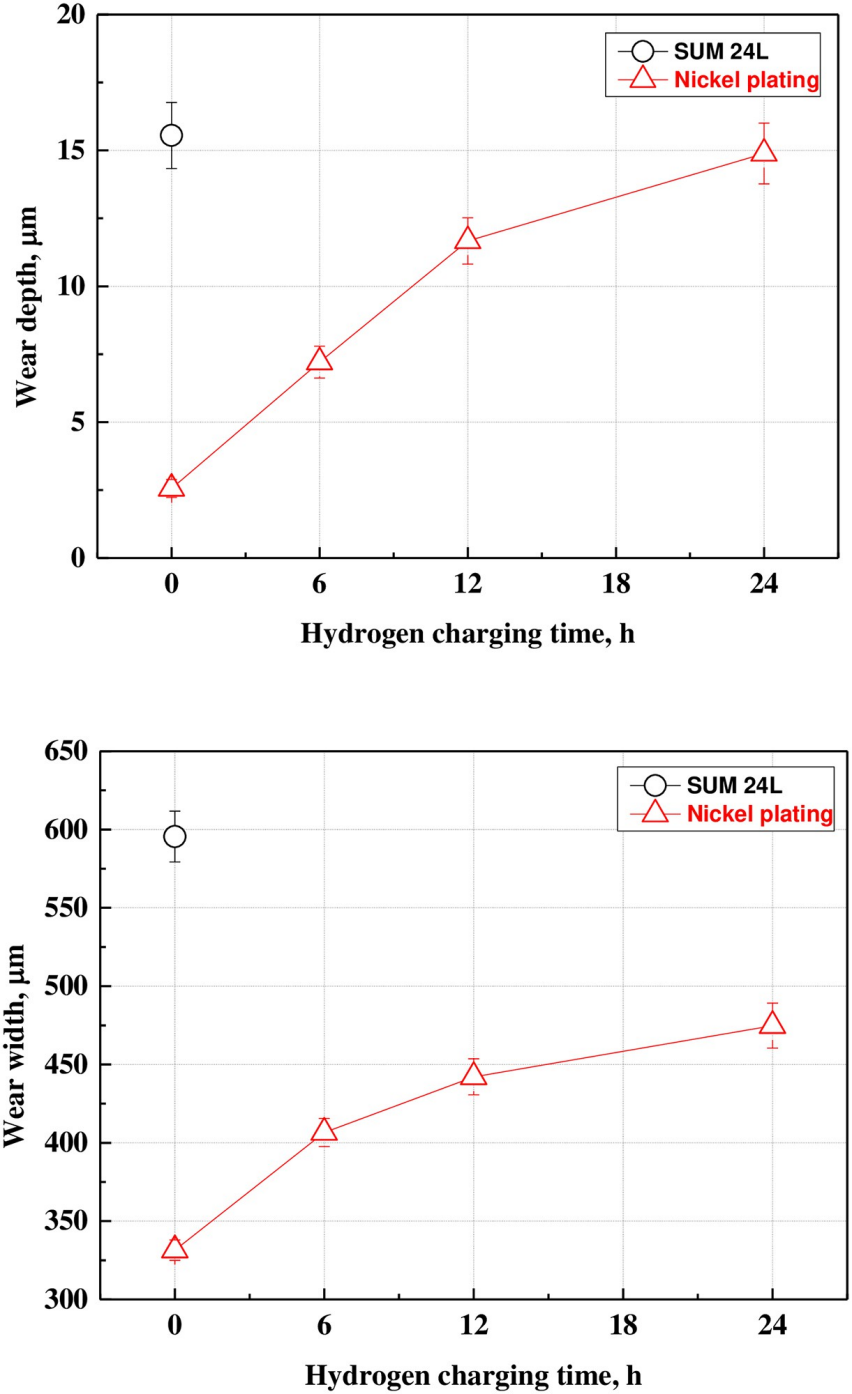
Wear depth and width of wear track after sliding friction experiment of electroless nickel-plated free-cutting steel and substrate with/without hydrogen permeation. (a) Wear depth, (b) Wear width.

Figs 11 and 12 present the results of measuring and analyzing the wear depth, width, arithmetic average roughness(Ra) and maximum roughness (Rmax) of the wear track with a 3D confocal laser microscope after the sliding wear experiment. The free-cutting steel exhibited an abrasive wear trend of the counter ball shape and had the largest wear depth (15.55 μm) and width (595.41 μm) among all samples. However, the wear depth and width of the electroless nickel plating layer before hydrogen permeation were 2.56 μm and 331.45 μm, respectively, indicating a significantly lower wear damage tendency compared with that of free-cutting steel. In addition, the wear track was dominated by abrasive wear in a flat shape. After hydrogen permeation for 24 h, the wear depth and width increased up to 14.67 μm and 474.79 μm, respectively, which increased by about 5.7 and 1.4 times compared with those of the electroless nickel plating sample before hydrogen permeation; the adhesive wear trend was also clearly observed. The arithmetic average roughness presented a similar trend. Free-cutting steel exhibited the largest Ra of 4.381 μm, about 4.9 times larger than the electroless nickel-plated specimen, which measured 0.883 μm. Furthermore, the Ra increased with increasing in hydrogen charging time up to 2.853 μm. Nevertheless, the Ra of the hydrogen-permeated electroless nickel plating was at least 34% lower than that of the free-cutting steel. In addition, Rmax was calculated after measuring the maximum peak height (Rp) and maximum valley depth (Rv). Rmax also increased with hydrogen charging time. However, unlike Ra, the Rmax value of the hydrogen permeated specimen for 24 hours was larger than that of the free-cutting steel. Since Rmax is calculated using Rp and Rv, it is believed that the value for the electroless nickel-plated specimen with relatively low uniformity was large. As a result, it can be confirmed that the wear resistance of the electroless nickel plating layer is reduced by hydrogen permeation. In addition, since the wear depth, width and Ra of the free-cutting steel are the largest, it is considered that the electroless nickel plating layer exhibited remarkably excellent wear resistance.

**Fig 13:**
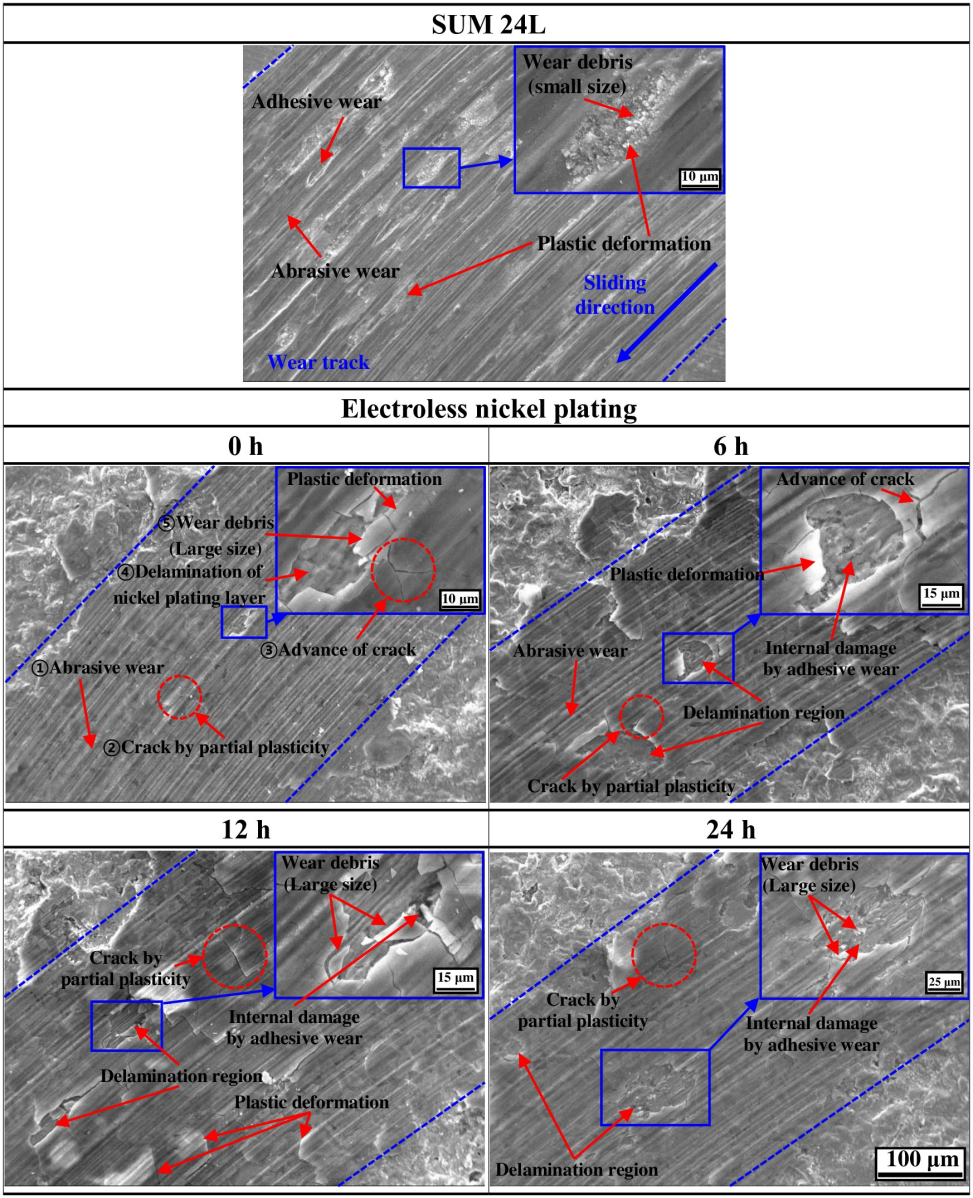
Wear track morphologies of SUM 24L and electroless nickel-plated free-cutting steel with/without hydrogen permeation.

Fig 13 demonstrates the wear track morphologies of free-cutting steel and electroless nickel-plated sample with FE-SEM after the sliding wear experiment. In free-cutting steel, the abrasive wear and localized-adhesive wear was observed together. In particular, wear debris and traces of plastic deformation were observed in areas where abrasive wear and adhesive wear occurred. In general, metal contact generates frictional heat during friction/wear, plasticizing the surface [71]. At this time, the plastically deformed area acts as an adhesive junction and causes adhesive wear [72]. In addition, crack and wear debris is formed due to plastic deformation of the surface, resulting in a tendency for three-body adhesive wear [73,74]. This is a factor that accelerates wear damage. In the electroless nickel-plated sample with 0 hours of hydrogen permeation, crack and localized delamination of the nickel plating surface were observed, and the abrasive wear was dominant. This wear tendency is thought to be caused by frictional heat that plasticizes the nickel plating layer. First, the nickel-plated surface forms wear tracks due to adhesive wear (①). Afterward, frictional heat is generated, partially plasticizing the surface of nickel plating layer, and forming cracks due to wear and shear force (②). Moreover, continuous wear and shear force cause the cracks to advance (③) and, in severe cases, delaminate the plastically deformed nickel plating layer (④). Additionally, relatively large-sized wear debris is formed (⑤). This wear trend was similar regardless of the hydrogen permeation time. Cracks and localized delamination of the nickel-plated surface were observed inside the wear track under all experimental conditions, and abrasive wear was dominant. However, as the hydrogen permeation time increased, the area where the nickel plating layer was delaminated increased. Additionally, internal damage due to substrate exposure and adhesive wear was observed more clearly. Compared to free-cutting steel, the size of wear debris in the nickel plating layer was significantly larger. As a result, the nickel plating layer acts as a barrier to improve hydrogen permeation resistance and wear resistance. However, continuous hydrogen permeation and friction/wear plastically deform the nickel plating layer, reducing durability. In addition, it is believed that large-sized wear debris is formed, which acts as a factor in accelerating the degradation of the sample.

## 4. Conclusion

In this research, the results of the experiment on the effect of hydrogen permeation on the mechanical characteristics of electroless nickel-plated free-cutting steel are as follows.

The surface of the electroless nickel plating layer was damaged and peeled off by hydrogen permeation. Due to the inherent characteristics of electroless nickel plating, the cracks and delamination of grain by hydrogen permeation were clearly observed at the grain boundaries and triple junctions. In particular, the cracks grew from grain boundary toward the intergranular. This is because the grain boundaries and triple junctions are hydrogen permeation pathways and increasing area of the hydrogen partial pressure. As a result, the surface roughness increased by a maximum of two times.

As a result of the indentation experiment, the Vickers hardness of the electroless nickel plating layer decreased from 719 HV to a maximum of 466 HV as the hydrogen permeation time increased. This is thought to be attributed to the decrease in hardness resulting from the relative decrease in bonding strength and compressive residual stress according to the surface damage of the nickel plating layer with hydrogen permeation.

The adhesion strength of the electroless nickel plating layer decreased to 17.1 N(0 h sample), 16.9 N(6 h sample), 15.5 N(12 h sample), and 13.9 N(24 h sample), respectively, as the hydrogen permeation time increased. In particular, the acoustic emission signal value measured during the scratch experiment rapidly decreased after 12 h or more of the hydrogen permeation experiment. This means that the adhesion strength and durability of the electroless nickel plating layer decreased as a result of hydrogen permeation.

In particular, hydrogen permeation increased the friction coefficient of the electroless nickel-plated layer, and the damage caused by adhesive wear was significantly greater, increasing the wear depth by up to 5.7 times. This is believed to be due to the decreasing in wear resistance of the electroless nickel plating layer damaged by hydrogen permeation. In particular, the micro-particles and wear debris formed by hydrogen permeation and friction/wear act as a third bodies and accelerate the wear damage of the material.

As a result, the surface roughness and friction coefficient of the electroless nickel-plated specimens damaged by hydrogen permeation increased, while the Vickers hardness and adhesion strength decreased. In addition, the electroless nickel-plated layer, which had relatively reduced durability, presented a significantly greater tendency for adhesive wear during friction/wear. In particular, the changes in surface roughness, Vickers hardness, and adhesion strength are closely related to the friction coefficient and wear tendency (abrasive and adhesive wear). Therefore, the above factors should be considered together to evaluate hydrogen permeation resistance.

Nevertheless, the Vickers hardness and the friction coefficient of the electroless nickel plating layer were improved by about 3 and 5.6 times, respectively, compared with those of the free-cutting steel. In particular, the electroless nickel-plated specimens with hydrogen embrittlement exhibited significantly better Vickers hardness and wear resistance than the free-cutting steel. As a result, electroless nickel plating is considered a suitable surface treatment technology for the hydrogen industry.
